# Phosphatidylinositol 5-Phosphate-Loaded Apoptotic Body-Like Liposomes for *Mycobacterium abscessus* Infection Management in Patients With Cystic Fibrosis

**DOI:** 10.1093/infdis/jiaf124

**Published:** 2025-04-18

**Authors:** Tommaso Olimpieri, Noemi Poerio, Fabio Saliu, Nicola I Lorè, Fabiana Ciciriello, Greta Ponsecchi, Marco M D’Andrea, Federico Alghisi, Daniela M Cirillo, Maurizio Fraziano

**Affiliations:** Department of Biology, University of Rome Tor Vergata, Rome, Italy; Department of Biology, University of Rome Tor Vergata, Rome, Italy; Emerging Bacteria Pathogens Unit, San Raffaele Scientific Institute, Milan, Italy; Emerging Bacteria Pathogens Unit, San Raffaele Scientific Institute, Milan, Italy; Pneumology and Cystic Fibrosis Unit, Bambino Gesù Children's Hospital, Scientific Institute for Research Hospitalization and Healthcare (IRCCS), Rome, Italy; Department of Biology, University of Rome Tor Vergata, Rome, Italy; PhD Program in Evolutionary Biology and Ecology, Department of Biology, University of Rome Tor Vergata, Rome, Italy; Department of Biology, University of Rome Tor Vergata, Rome, Italy; Pneumology and Cystic Fibrosis Unit, Bambino Gesù Children's Hospital, Scientific Institute for Research Hospitalization and Healthcare (IRCCS), Rome, Italy; Emerging Bacteria Pathogens Unit, San Raffaele Scientific Institute, Milan, Italy; Department of Biology, University of Rome Tor Vergata, Rome, Italy

**Keywords:** *Mycobacterium abscessus*, cystic fibrosis, liposomes, ETI, host-directed therapy, pathogen-directed therapy, innate immunity, phosphatidylinositol 5-phosphate

## Abstract

The study investigates therapeutic strategies for managing chronic *Mycobacterium abscessus* infections, particularly in people with cystic fibrosis (PWCF) who are ineligible for standard elexacaftor, tezacaftor, ivakaftor (ETI) treatments. Apoptotic body-like liposomes loaded with phosphatidylinositol 5-phosphate (ABL/PI5P) were tested in vitro in *M. abscessus*-infected macrophages from PWCF as potential treatment. ABL/PI5P reduced intracellular bacterial viability and showed enhanced effects on a *M. abscessus* clinical strain when combined with amikacin. Notably, ABL/PI5P was effective on macrophages from PWCF not receiving ETI therapy. The findings suggest ABL/PI5P liposomes as a promising alternative or adjunct therapy, especially for those who cannot access ETI treatment, warranting further clinical investigation.

## Teaser

ABL/PI5P enhances anti-*Mycobacterium abscessus* response of macrophages from people with CF, receiving or not ETI therapy. Our results support the possible development of ABL/PI5P treatment as host-directed therapy against *M. abscessus* infection, especially in people with CF who cannot access ETI treatment.

Cystic fibrosis (CF) is an autosomal recessive genetic disease, caused by mutations in the gene encoding the CF transmembrane conductance regulator channel (CFTR) [1]. The pathophysiological changes in CF result in a systemic disease affecting the function of several organs, although the predominant cause of morbidity and mortality is severe progressive pulmonary disease caused by recurrent and chronic respiratory infections. In this context, *Mycobacterium abscessus*, an intrinsic drug-resistant microorganism requiring long-term administration of multiple antibiotics, has emerged as a significant pathogen in people with CF (PWCF), and infections sustained by this species are linked to disease progression and poor prognosis. The success rate of *M. abscessus* pulmonary disease treatment is still unsatisfactory and important adverse effects are common due to the long-term antibiotic combination therapy [2].

A defective antimicrobial response exerted by innate immune cells in PWCF has been reported, in part due to a dysfunctional phagocytosis process [1], which is regulated by the expression of secondary lipid messengers, such as phosphatidylinositol 5-phoshate (PI5P), which can mediate phagosome acidification and noncanonical autophagy processes when delivered inside cells by apoptotic body-like liposomes (ABL) [3]. In this context, we have shown that bronchoalveolar lavage cells from patients with drug-resistant pulmonary infections increased significantly their capacity to kill in vivo acquired bacterial pathogens when ex vivo stimulated with ABL/PI5P [3]. Moreover, intranasal administration of ABL/PI5P, in wild-type and CF mice infected with *M. abscessus* resulted in a significant reduction of both pulmonary inflammation and mycobacterial burden, and the combined treatment with ABL/PI5P plus amikacin resulted in a higher reduction of both parameters in comparison with single treatments [4].

The introduction of CFTR modulators, able to restore CFTR function, has significantly improved the clinical course and management of PWCF. In particular, ETI, the triple-combination consisting of 2 CFTR correctors (elexacaftor, tezacaftor) and 1 CFTR potentiator (ivakaftor), grants a significant improvement of lung function and quality of life in PWCF [5]. Its early administration may reduce airway infections, although there could be patient-to-patient variability in the therapy outcome [6]. However, not all PWCF can receive ETI therapeutic regimen, as it excludes several CFTR mutations, underscoring the urgent need for the development of novel therapeutic strategies [5]. On these grounds, the aim of this study is to evaluate the in vitro immunotherapeutic potential of ABL/PI5P, in combination with either ETI or amikacin, on *M. abscessus*-infected macrophages from PWCF receiving or not ETI.

## METHODS

### Ethics Statement

PWCF, giving their (or parental) written informed consent to participate in the study, were enrolled at Bambino Gesù Children's Hospital in Rome after having received detailed information on the scope and objectives of the study by medical personnel, who explained the patient information leaflet (ethics approval No. 738/2017 of Bambino Gesù Children's Hospital, Rome).

### Patients

PWCF (n = 36) were enrolled at Bambino Gesù Children's Hospital in Rome, Italy. All PWCF were clinically stable at the time of blood donation (5 mL). Clinical and demographic features of PWCF are summarized in Supplementary Tables 1 and 2. Primary monocyte-derived macrophages (MDM) from PWCF were prepared as previously described [6].

### Liposome Preparation

ABLs carrying 1,2-dioleoyl-sn-glycero-3-phospho (1′-myo-inositol-5′-phosphate) (PI5P; Avanti Polar Lipids) were produced and quantified as previously described [4].

### Bacteria

*M. abscessus* reference strain American Type Culture Collection (ATCC) 19977 and the *M. abscessus* subsp *abscessus* clinical strain Mab285 (previously referred to as CP07), a representative of the dominant clone 1 of *M. abscessus* subsp *abscessus*, were used [7]. *M. abscessus* and Mab285 single colonies were collected by streaking on Middlebrook 7H10 medium (BD Difco) supplemented with oleic acid, albumin, dextrose, and catalase, then suspended in 15 mL of Middlebrook 7H9 broth (BD Difco) supplemented with albumin, dextrose, catalase, and 0.05% Tween 80, and grown in Erlenmeyer flask at 37° C with stirring for 40 hours. Growth was monitored by measuring the optical density at wavelength 600 nm by a spectrophotometer (Varioskan LUX Multimode Microplate Reader; Thermo Fisher Scientific).

### Evaluation of In Vitro Bacterial Extracellular/Intracellular Growth

To assess the intracellular bacterial growth, MDM from PWCF were prestimulated with elexacaftor 5 µM plus tezacaftor 5 µM plus ivacaftor 1 µM (ETI) for 2 days and then infected with *M. abscessus*, for 3 hours at 37° C at a multiplicity of infection (MOI) of 10. Thereafter, extracellular bacilli were killed by 1 hour incubation with 250 µg/mL amikacin. Cells were then washed and incubated with ABL/PI5P (ratio 1:1) and/or ETI for 18 hours. Finally, cells were lysed with 1% deoxycholate (Sigma), diluted in phosphate-buffered saline-Tween 80 and colony-forming units (CFU) were quantified by plating bacilli in triplicate on 7H10. To evaluate the in vitro efficacy of a combined therapy on extracellular and intracellular mycobacterial viability, MDM from PWCF were infected with Mab285 at a MOI of 10 for 3 hours at 37° C. Cells were then stimulated with ABL/PI5P and/or 4 µg/mL amikacin for 18 hours. Both extracellular and intracellular bacterial growth were assessed by plating on 7H10 agar.

### Statistics

Statistical significance was determined by 2-sided Wilcoxon rank sum test.

## RESULTS

### ABL/PI5P Reduces *M. abscessus* Intracellular Viability in Macrophages From PWCF Irrespective of ETI Therapeutic Regimen Eligibility

**Figure 1.**
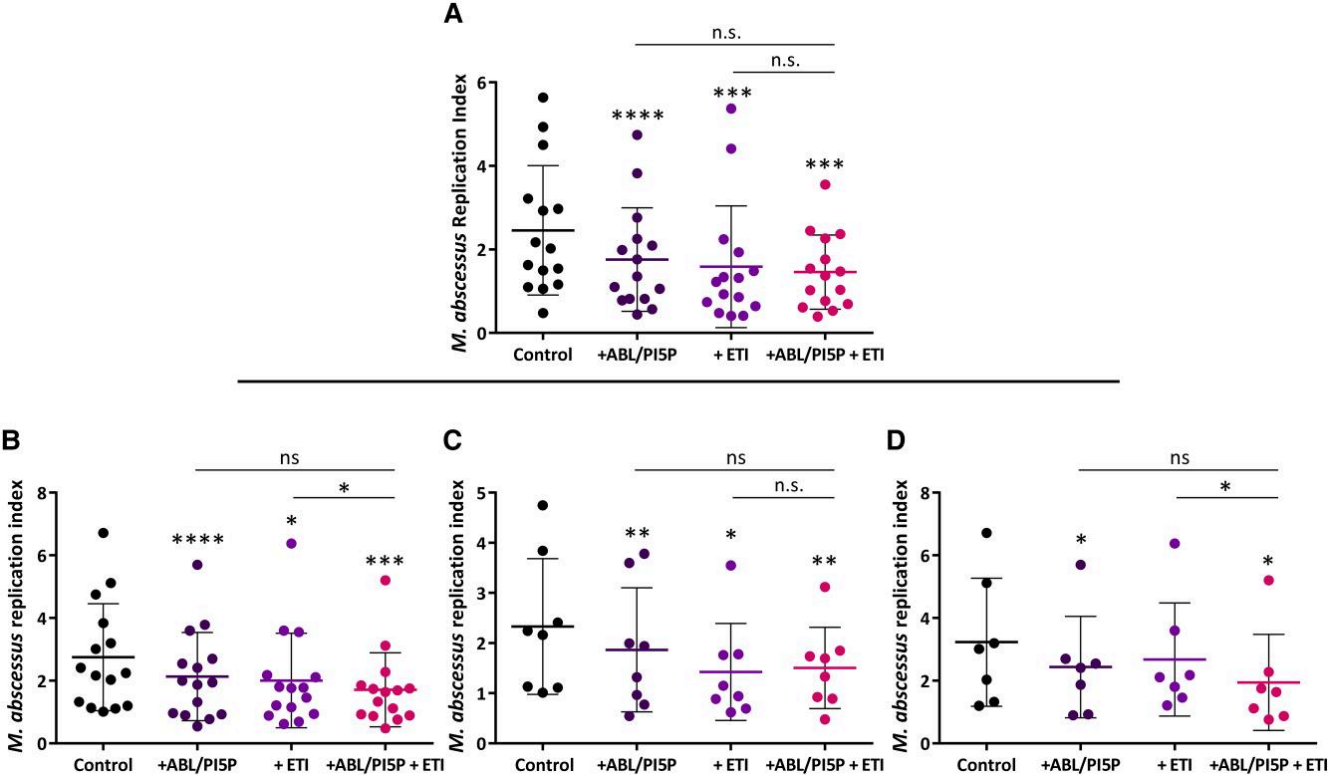
ABL/PI5P reduces *Mycobacterium abscessus* intracellular viability in macrophages from PWCF irrespective of ETI therapeutic regimen eligibility. Monocyte-derived macrophages from PWCF under an ETI regimen (n = 15) (*A*) or from PWCF without a modulator regimen (n = 15) (*B*) were cultured at a concentration of 1 × 10^6^ cells/mL in 96-well plates. Cells were then infected with *M. abscessus* at multiplicity of infection 10 for 3 hours and finally stimulated with ABL/PI5P and/or ETI for 18 hours. Replication index was calculated as the ratios between the colony-forming units obtained after 18 hours from infection, in the presence or absence of stimuli, and those obtained immediately after infection. For a deeper analysis of the results, patients with CF represented in (*B*) were divided into 2 groups: (*C*) those eligible (n = 8) and (*D*) not eligible (n = 7) for the ETI therapeutic regimen. The results are shown as mean ± standard deviation of the values obtained. Statistical analysis was performed by 2-sided Wilcoxon matched-pairs signed rank test. *P < .05, **P < .01 ***P < .001, ****P < .0001. If not indicated by the line, the comparisons were performed versus control. Abbreviations: ABL/PI5P, apoptotic body-like liposome/phosphatidylinositol 5-phosphate; PWCF, people with cystic fibrosis; ETI, elexacaftor, tezacaftor, ivacaftor; ns, not significant.

To assess the impact of ETI on the ABL-induced antimycobacterial response, MDM from PWCF receiving ETI regimen (Supplementary Table 1) were infected in vitro with *M. abscessus* and then treated with ABL/PI5P and/or ETI. Figure 1*A* shows that both single and combined treatments significantly enhanced intracellular *M. abscessus* clearance in comparison to control, and that no additive, synergic, or interference effect was observed when ABL/PI5P and ETI were administrated in combination compared to single treatments.

The in vitro efficacy of the ABL/PI5P and/or ETI treatments in MDM from PWCF who do not receive ETI therapeutic regimen was also evaluated. Figure 1*B* shows that both single and combined treatments were capable of significantly reducing *M. abscessus* intracellular viability. As the in vitro treatment with ETI alone significantly decreased *M. abscessus* intracellular viability, we subdivided this latter group of patients into 2 categories: eligible and noneligible for ETI therapeutic regimen (Supplementary Table 2). In particular, the eligible subgroup comprised all those patients who (1) possess the F508del mutation and do not receive ETI therapeutic regimen because of age, treatment refusal, or waiting for the drug prescription; (2) have a mutation considered eligible for the ETI regimen in only the United States [8]; and (3) have a mutation that is currently under trial in Europe (European Union Drug Regulating Authorities Clinical Trials Database [EudraCT] number 2021-005914-33). The noneligible subgroup comprised PWCF whose mutations are considered incompatible with the ETI therapeutic regime both in the United States and European Union (EU).

ETI in vitro treatment of macrophages from eligible patients resulted in a significant reduction of *M. abscessus* intracellular viability (Figure 1*C*), whereas no statistically significant effect was observed in the noneligible subgroup, suggesting that ETI could be ineffective in terms of recovery of antimicrobial response in these patients (Figure 1*D*). Importantly, ABL/PI5P treatment reduced *M. abscessus* intracellular viability in macrophages of PWCF without a modulator therapeutic regimen, irrespective of their eligibility status for the drugs (Figure 1*C* and 1*D*).

### ABL/PI5P-Amikacin Combined Treatment Reduces MAb285 Intracellular Viability in Macrophages of PWCF Who do Not Receive ETI Therapeutic Regimen

**Figure 2.**
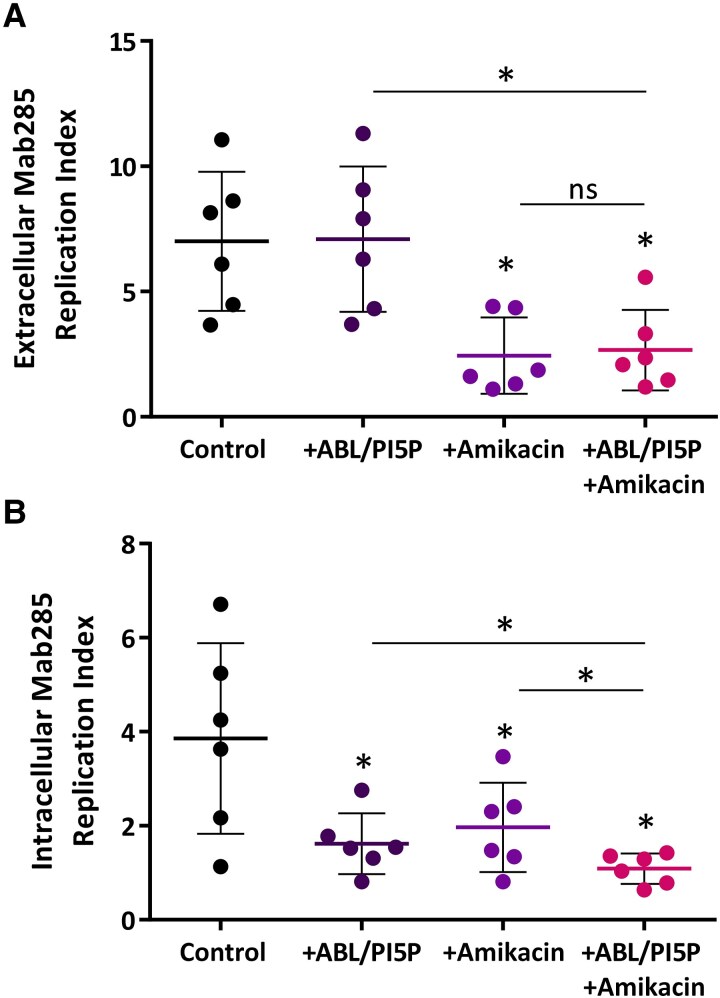
ABL/PI5P-amikacin combined treatment reduces Mab285 intracellular viability in macrophages of PWCF who do not receive ETI therapeutic regimen. MDM from PWCF without a modulator regimen (n = 6) were cultured at a concentration of 1 × 10^6^ cells/mL in 96-well plates. Cells were then infected with *Mycobacterium abscessus* at multiplicity of infection 10 for 3 hours and stimulated with ABL/PI5P and/or 4 µg/mL amikacin for 18 hours. Finally, supernatant was collected, and MDM were lysed to enumerate extracellular (*A*) and intracellular (*B*) bacteria. Replication index was calculated as the ratio between the colony-forming units obtained 18 hours after infection in the presence or absence of ABL/PI5P and/or amikacin, and those obtained immediately after infection and before the stimuli. The results are shown as mean ± standard deviation of the values obtained. Statistical analysis was performed by 2-sided Wilcoxon matched-pairs signed rank test. *P < .05. If not indicated by the line, the comparisons were performed versus control. Abbreviations: ABL/PI5P, apoptotic body-like liposome/phosphatidylinositol 5-phosphate; PWCF, people with cystic fibrosis; ETI, elexacaftor, tezacaftor, ivacaftor; MDM, monocyte-derived macrophages; ns, not significant.

The combined treatment ABL/PI5P-amikacin promotes a higher reduction of intracellular *M. abscessus* viability in CF macrophages compared to single treatments [4]. As a combined therapy, based on antibiotic and bioactive liposomes, may represent a valuable strategy to differentially target extracellular and intracellular pathogens, we tested its efficacy in improving the mycobactericidal activity in Mab285-infected CF macrophages from patients who are not receiving ETI (Supplementary Table 2). Results in Figure 2 show that ABL/PI5P did not show any direct effect on the extracellular pathogen (Figure 2*A*), whereas it significantly reduced the intracellular Mab285 viability (Figure 2*B*). Furthermore, the combined treatment with ABL/PI5P and amikacin (Figure 2*B*) induced a significant higher reduction of intracellular Mab285 replication index than single treatments.

## DISCUSSION

PWCF are subjected to chronic infections, including those caused by *M. abscessus*, which are linked to high morbidity and mortality rates [2]. According to the international guidelines for *M. abscessus* treatment, therapy requires a prolonged intravenous administration of multiple antibiotics, which can last years and are associated with significant toxicity and a high rate of treatment failures [2, 9]. In the era of CFTR modulators, ETI administration can improve clinical outcomes, reducing both (myco)bacterial infections and pulmonary exacerbations in PWCF [10].

In the United States, ETI can be administered to PWCF carrying at least 1 copy of the F508del *CFTR* mutation, or at least 1 of the 177 approved rare mutations, supported by in vitro data [8]. In the EU, ETI is approved and prescribed only to PWCF carrying at least 1 copy of the F508del mutation. ETI administration clinical trials have been conducted for just 18 rare variants (EudraCT No. 2021-005914-33), but ETI usage is not yet approved [11]. According to US or EU guidelines ETI can be prescribed to patients of at least 2 years of age, but in Italy, where this study was conducted, national legislation limits ETI administration to patients older than 6 years. In addition, not all PWCF can benefit from this modulator due to their genetic variants and the high cost of the drug, highlighting the need for alternative approaches [5].

An altered antimicrobial response exerted by innate immune cells in PWCF may depend, at least in part, on a dysfunctional phagocytosis process [12, 13]. In this context, we have previously reported a host-directed therapeutic strategy composed of bioactive liposomes carrying ABL/PI5P, able to enhance phagosome-dependent antimicrobial response irrespective of the pathogen's antibiotic resistance [3]. Here, we show that in macrophages from ETI-eligible PWCF either under the therapeutic regimen or not, the in vitro combined treatment ABL/PI5P-ETI decreases *M. abscessus* intracellular viability, although not significantly when compared to single treatments (Figure 1*A* and 1*C*), suggesting lack of interference of ABL/PI5P with ETI during in vitro treatment. In addition, we demonstrated that ABL/PI5P single treatment promotes mycobactericidal activity in macrophages of PWCF receiving ETI (Figure 1*A*) or not receiving ETI (Figure 1*B*), regardless of their eligibility (Figure 1*C* and 1*D*). The extent of in vitro treatment efficacy with ABL/PI5P reported here is consistent with our previously reported results obtained in macrophages from PWCF, and although the reduction in intracellular mycobacterial viability may appear moderate, it translates in vivo into a 50-fold reduction in pulmonary mycobacterial burden following intranasal treatment in *M. abscessus*-infected CF mice [4]. In addition, the present study shows that ABL/PI5P-amikacin combined treatment promotes a higher reduction of the Mab285 clinical strain in CF macrophages for patients not receiving ETI in comparison with single treatment, suggesting the possibility to combine the host-directed with the pathogen-directed therapeutic approach to simultaneously target both intracellular and extracellular *M. abscessus*. Altogether, these results support the possible development of ABL/PI5P treatment as an alternative or adjunct therapy for PWCF, especially for those (about 10% in the United States and about 30% in Italy [14, 15]) who cannot benefit from ETI treatment.

## Supplementary Material

jiaf124_Supplementary_Data

## Supplementary Data

Supplementary materials are available at *The Journal of Infectious Diseases* online (http://jid.oxfordjournals.org/). Supplementary materials consist of data provided by the author that are published to benefit the reader. The posted materials are not copyedited. The contents of all supplementary data are the sole responsibility of the authors. Questions or messages regarding errors should be addressed to the author.

## Notes

***Acknowledgments.*** We thank Carla Viscomi, head nurse of the Cystic Fibrosis Unit, Pediatric Hospital Bambino Gesù in Rome, for her important support in the management and collection of clinical samples from patients.

***Financial support.*** This work was supported by the Italian Cystic Fibrosis Research Foundation (FFC) (Delegazione FFC Ricerca di Ascoli Piceno; Gruppo di Sostegno FFC Ricerca di Saviano; Delegazione FFC Ricerca di Padova; Delegazione FFC Ricerca di Latina; Delegazione FFC Ricerca di Milano; Gruppo di Sostegno FFC Ricerca di Genova Mamme per la Ricerca) (grant number FFC 13/2022); and by Rome Technopole, a National Recovery and Resilience Plan (PNRR) project (grant number ESC00000024 to N. P.).
