# Supplementary material for: Phosphatidylinositol 5-Phosphate-Loaded Apoptotic Body-Like Liposomes for Mycobacterium abscessus Infection Management in Patients With Cystic Fibrosis
Source: J Infect Dis. 2025 Apr 18;232(1):e43–7. doi: 10.1093/infdis/jiaf124 (PMC12308664; doi:10.1093/infdis/jiaf124)
Supplement: jiaf124_Supplementary_Data [file jiaf124_supplementary_data.zip › Supplementary_Table_S1.docx]

**Phosphatidylinositol 5-phosphate loaded apoptotic body-like liposomes for *Mycobacterium abscessus* infection management in cystic fibrosis patients**

Tommaso Olimpieri^1*^, Noemi Poerio^1*^, Fabio Saliu^2^, Nicola I. Lorè^2^, Fabiana Ciciriello^3^, Greta Ponsecchi^1,4^, Marco M. D’Andrea^1^, Federico Alghisi^3^, Daniela M. Cirillo^2^, and Maurizio Fraziano^1 °^

^1^Dept. of Biology, University of Rome Tor Vergata, 00133, Rome, Italy.
^2^Emerging Bacteria Pathogens Unit, San Raffaele Scientific Institute, 20132, Milan, Italy.

^3^Pneumology and cystic fibrosis unit, Bambino Gesù Children's Hospital, IRCCS, 00165 Rome, Italy

^4^PhD Program in Evolutionary Biology and Ecology, Dept. of Biology, University of Rome Tor Vergata, 00133, Rome, Italy.

**Supplementary Table 1. Demographic and clinical characteristics of pwCF under ETI treatment.**

**
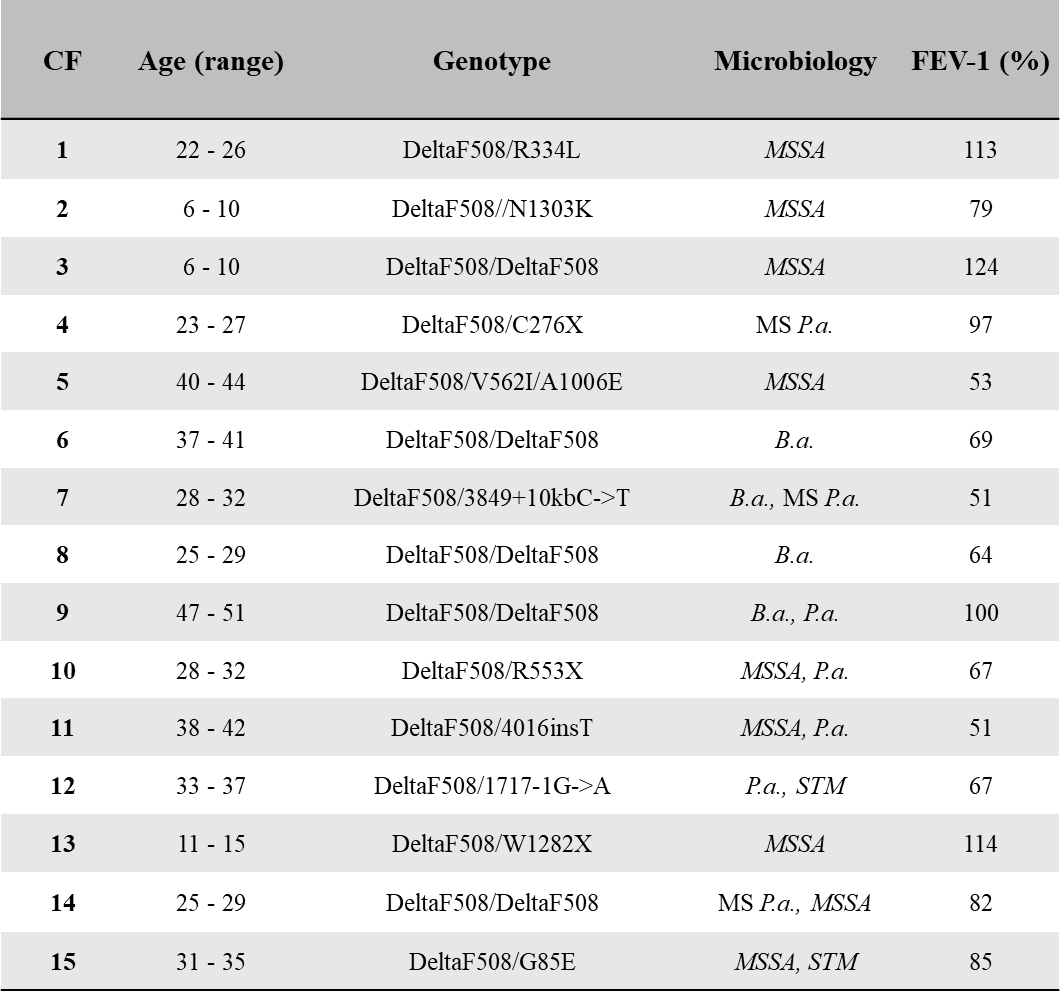
**

Data are related to patients analysed in Figure 1A. *Abbreviations: B.a.: Branhamella catarrhalis; MS P.a.: Pseudomonas aeruginosa* Mucoid Strain*; MSSA: Methicillin-sensitive Staphylococcus aureus; P.a.: Pseudomonas aeruginosa; STM: Stenotrophomonas maltophilia.*
