# Supplementary material for: Phosphatidylinositol 5-Phosphate-Loaded Apoptotic Body-Like Liposomes for Mycobacterium abscessus Infection Management in Patients With Cystic Fibrosis
Source: J Infect Dis. 2025 Apr 18;232(1):e43–7. doi: 10.1093/infdis/jiaf124 (PMC12308664; doi:10.1093/infdis/jiaf124)
Supplement: jiaf124_Supplementary_Data [file jiaf124_supplementary_data.zip › Supplementary_Table_S2.docx]

**Phosphatidylinositol 5-phosphate loaded apoptotic body-like liposomes for *Mycobacterium abscessus* infection management in cystic fibrosis patients**

Tommaso Olimpieri^1*^, Noemi Poerio^1*^, Fabio Saliu^2^, Nicola I. Lorè^2^, Fabiana Ciciriello^3^, Greta Ponsecchi^1,4^, Marco M. D’Andrea^1^, Federico Alghisi^3^, Daniela M. Cirillo^2^, and Maurizio Fraziano^1 °^

^1^Dept. of Biology, University of Rome Tor Vergata, 00133, Rome, Italy.
^2^Emerging Bacteria Pathogens Unit, San Raffaele Scientific Institute, 20132, Milan, Italy.

^3^Pneumology and cystic fibrosis unit, Bambino Gesù Children's Hospital, IRCCS, 00165 Rome, Italy

^4^PhD Program in Evolutionary Biology and Ecology, Dept. of Biology, University of Rome Tor Vergata, 00133, Rome, Italy.


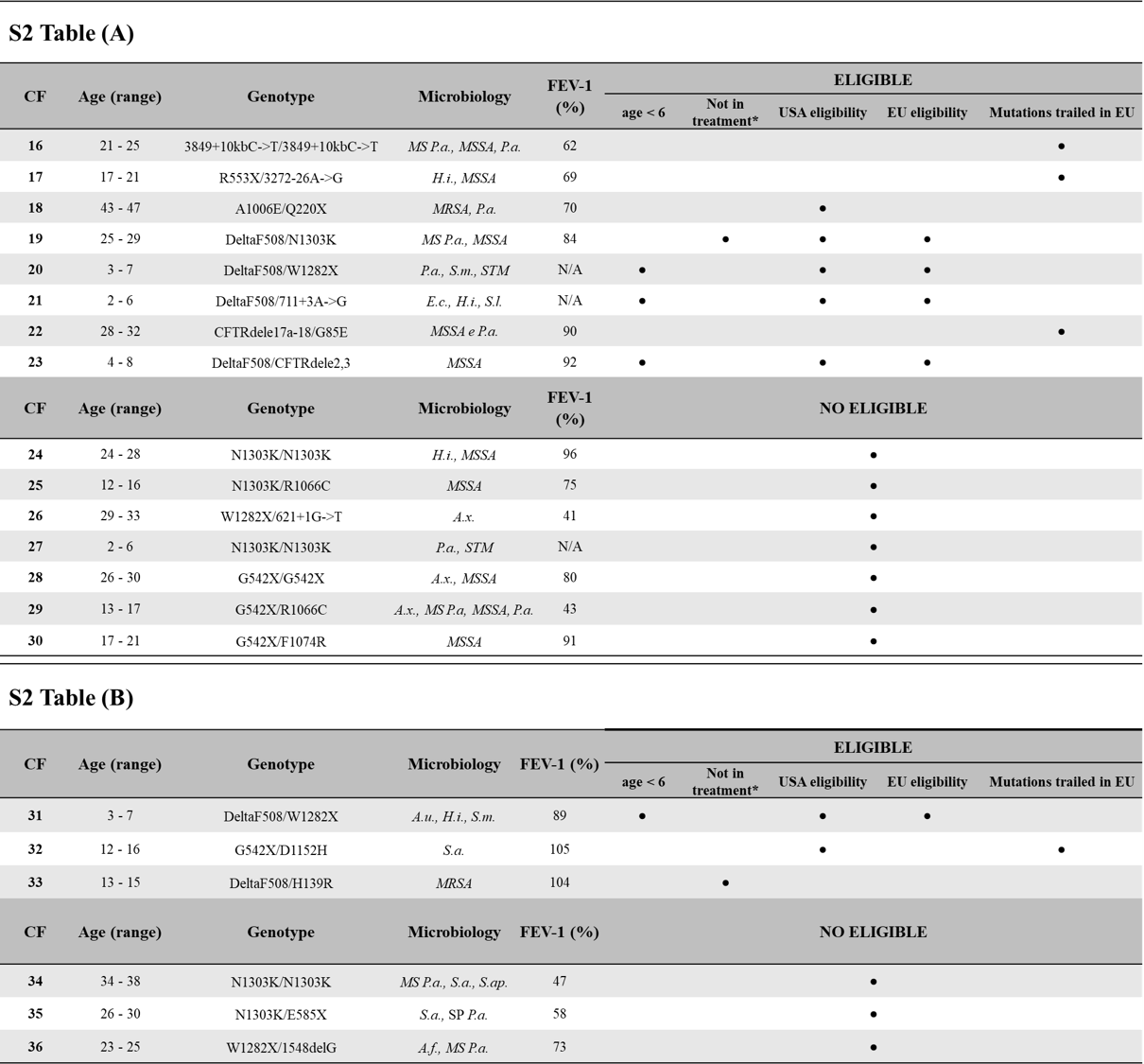
**Supplementary Table 2. Demographic and clinical characteristics of pwCF not receiving ETI treatment.**

(A) Data are related to patients analysed in Figure 1B-D. (B) Data are related to patients analysed in Figure 2. *Abbreviations: A.f.: Aspergillus fumigatus; A.u.: Acinetobacter ursingii; A.x.: Achromobacter xylosoxidans; E.c.: Escherichia coli; H.i.: Haemophilus influenzae; MS P.a.: Pseudomonas aeruginosa* Mucoid Strain*; MSSA: Methicillin-sensitive Staphylococcus aureus; MRSA: Methicillin-resistant Staphylococcus aureus; P.a.: Pseudomonas aeruginosa; S.ap.:* *Scedosporium apiospermum; S.l.: Serratia liquefaciens; S.m.: Serratia marcescens; S.a.: Staphylococcus aureus; SP P.a.: Pseudomonas aeruginosa* Small Phenotype*; STM: Stenotrophomonas maltophilia;* N/A: no applicable: CF patients unable to safely perform spirometry because of their age; not in treatment*: eligible CF patients for ETI regimen either waiting for the prescription or refusing the medication. Patients listed as 20 and 31 are the same CF patient who did two blood donations one year apart.
